# Interventions to Improve Body Composition, Upper and Lower Extremity Muscle Strength, and Balance Ability of Older Female Adults: An Intervention Study

**DOI:** 10.3390/ijerph19084765

**Published:** 2022-04-14

**Authors:** Wei-Yang Huang, Cheng-En Wu

**Affiliations:** 1 Physical Education, National Taiwan College of Performing Arts, Taipei 11464, Taiwan; pmp999@tcpa.edu.tw; 2 Ph.D. Program of Technology Management, Chung Hua University, Hsinchu 30012, Taiwan

**Keywords:** elderly female, moderate-intensity physical activity, sarcopenia, body composition, interventions

## Abstract

The aim of the present study was to understand the effects of a moderate-intensity physical activity program on the changes observed in the body composition, upper and lower extremity muscle strength, as well as balance in elderly female adults in order to evaluate sarcopenia. In this study, 30 healthy elderly females were recruited and were randomly assigned to either the control group or the experimental group. The experimental group engaged in a moderate-intensity physical activity program twice a week for 8 weeks. Using a body composition analyzer, the senior fitness test, and handgrip strength and gait speed tests, all participants were tested in pre- and post-tests. The results of the study revealed changes in the overall body composition in the experimental group, with significant decreases in body mass index, body fat percentage, and body fat mass and substantial increases in the basal metabolic rate and skeletal muscle mass, while the upper and lower extremity muscle strength and balance ability also showed significant improvements. The moderate-intensity physical activity program also increased upper limb handgrip strength and lower limb gait speed, showing that the plan was able to effectively evaluate sarcopenia. The study concluded that using upper limb handgrip strength and lower limb walking speed to evaluate sarcopenia are useful diagnostic tools. Moderate-intensity physical activity is effective for improving muscle strength and reducing sarcopenia.

## 1. Introduction

In 2010, The European Working Group on Sarcopenia in Older People (EWGSOP) initially defined sarcopenia as low muscle mass [1]. The EWGSOP updated this definition in 2019, further emphasizing that low muscle strength is also a key feature of sarcopenia and agreed that low muscle mass must be combined with low muscle function in the definition of sarcopenia [2]. In short, sarcopenia is a condition in which the skeletal muscle mass is low and the body has low functional muscle strength to perform activities of daily living (ADL) on its own. Muscle growth reaches its maximum strength at around the age of 30, but as we age, we lose about 3–5% of our strength every 10 years [3]. Sarcopenia is a common problem in the elderly, with there being a 10–26% chance of developing sarcopenia [4]. Sarcopenia may affect up to 80% of the elderly over the age of 80 [5]. One of the causes of sarcopenia is the degeneration of the skeletal muscles in the elderly along with a lack of exercise and a low protein intake, which can lead to aging-related diseases over time [6]. Seniors with sarcopenia are at a higher risk of developing serious co-morbidities, while other diseases will further reduce their mobility, physical function, and independence [7].

Regarding the relationship between body composition and sarcopenia, first of all, it is necessary to understand that body composition includes body fat mass, body fat percentage, skeletal muscle, and basal metabolic rate [8]. Cunningham determined the relationship between basal metabolic rate (BMR) and skeletal muscle mass (SMM) and body fat mass (BFM) from a physiological point of view [9] and pointed out that females lose muscle mass and strength earlier than males, and that the loss of muscle mass observed in females is accelerated during menopause [10]. In addition, changes in body composition are also associated with age and sedentary lifestyle, with declines in muscle mass and function being observed with age [11]. Therefore, the body composition of older females is associated with sarcopenia. Secondly, regarding the relationship between upper and lower extremity muscle strength and sarcopenia, according to a study by the EWGSOP, gait speed (GS) and/or hand-grip strength (HGS) are the simplest and most reliable methods of measuring sarcopenia [2]. As gait is the dynamic process of walking, the risk of developing sarcopenia is higher as one’s walking speed becomes slower. Farrell et al. also concluded that poor gait could be associated with a lack of exercise [12]. López-Teros et al. used gait speed and handgrip strength tests and determined that severe sarcopenia was associated with decreased balance and an increased risk of falls in older adults [13]. Franzon et al. suggested that gait speed and handgrip strength or chair stand tests are ways to assess sarcopenia in the elderly [14]. Finally, regarding the relationship between balance ability and sarcopenia, Kato et al. pointed out that the elderly have low muscle mass and low physical function and determined that older females are more likely to experience a decline in balance ability compared to older males [15]. Gadelha et al. demonstrated that severe sarcopenia affects balance and increases the risk of falls in older adults [16]. Cho et al. confirmed that lower extremity strength is associated with a risk of falls and balance in the elderly [17].

Studies have found that moderate physical activity in the elderly can help stimulate their bodies [18,19,20], enhance muscle strength to produce real benefits, and improve overall muscle fitness. Proper muscle strength exercises can prevent injuries and slow down the degeneration of the body as well as avoid the symptoms of muscle loss. Jefferis et al. showed that regular exercise is the most effective method for promoting physical health in older adults. With regular exercise, good muscle mass and muscle strength can be maintained without relying on others for daily activities [21]. Bardstu studied indicators of improved physical function and leg strength in older adults in the community after 8 months of resistance training [22]. A study by Chen et al. confirmed that physical activity in older adults can effectively improve physiological function by engaging in moderate to vigorous exercise, including aerobic exercise and strength training, at least five days per week and by participating in high levels of exercise [23]. According to all of the literature mentioned above, healthy elderly people can maintain good physiological function by participating in moderate-intensity aerobic- or resistance-based physical activities one or more times per week. As for the intensity of physical activity, it is generally assessed by the Metabolic Equivalent (MET), which is an internationally accepted measure [24]. The intensity of physical activity in this study was at a moderate intensity level, about 3.0–5.9 METs. It made people feel a little tired. Their breathing and heart rate were faster than usual, and they sweated a little. In terms of the type of physical activity, muscle strengthening activities and balance activities were adopted, and the Physical Activity Guidelines for Americans were applied [25]. The F.I.T.T. principles of frequency, intensity, time, and type of activity were used to design a complete physical activity program [26].

Based on the above literature, the purpose of this study was to intervene with a moderate-intensity physical activity program to improve the body composition, upper and lower extremity strength, and balance ability of elderly females to evaluate sarcopenia. Therefore, the first hypothesis (H1) of this study was that moderate-intensity physical activity would improve body composition in the participants. Hypothesis two (H2) was that moderate-intensity physical activity would improve upper- and lower-extremity muscle strength in the participants. Hypothesis three (H3) was that moderate intensity physical activity would improve the balance ability in the participants. Hypothesis four (H4) was that following this prescribed training program could optimize upper- and lower-extremity muscle strength and balance in those with evaluated sarcopenia.

## 2. Materials and Methods

### 2.1. Research Subjects

The study openly recruited 30 healthy elderly females from the Chung Hwa University human factor engineering laboratory, and only participants were surveyed for basic information, such as age, height, weight, etc. (excluding those who lacked adequate decision-making ability due to age, intelligence, or physical condition or those who were vulnerable to undue influence and coercion or who were unable to participate due to their environment, identity, or social and economic conditions) who were able make the decision of their own free will. The participants were randomly assigned to either the control group (mean age 72.1 ± 5.7 years) or the experimental group (mean age 71.8 ± 6.1 years). The two groups were assigned to complete the mean *t*-test for age (*t* = −0.74, *p* < 0.05), height (*t* = 0.61, *p* < 0.05), and weight (*t* = 0.83, *p* < 0.05). There were no significant differences in the pre-test *t*-test results between the above two groups, indicating that the results of random allocation of participants were homogeneous, as shown in Table 1. The participants in both groups were non-smokers, free of orthopedic or cardiac disease, and able to perform daily activities without assistance. The elderly females had never participated in a muscle training program before, but they were more physically active than sedentary people by engaging in weekly physical activities such as walking, gardening, cleaning windows and doors, vacuuming, washing cars, mopping, and keeping the yard organized. Each participant signed a consent form, and tests were conducted in a public setting. The results were anonymized (no information collected to identify a specific individual), the tests were non-invasive, and the protocols were approved by the Institutional Review Board of the Taipei City Hospital Renai Branch (approval number 110–86). The experiments were conducted in accordance with scientific and ethical principles. The participants in the control group, on the other hand, did not join this program. They lived as usual during the eight-week test period.

### 2.2. Research Materials

A moderate-intensity physical activity program was developed based on the Physical Activity Guidelines for Americans (PAGA) [25]. The participants in the experimental group engaged in activity four times per week, with moderate intensity meaning about 200 kcal were expended each time (5 METs × ⅔ h × 60). Moderate intensity was defined as the participant’s heart rate being between 64% and 76% of their maximum heart rate. The program ran for 8 weeks. The average attendance rate was 95%, with all participants in the experimental groups participating in 90% or more of the sessions. The physical activity program lasted for 8 weeks, with each activity being performed in a cycle of 6 movements of 1 min each (about 10–20 times) for a total of 3 cycles. The program included step-ups, chair squats, pistol squats, standing lunges, high leg lifts, 5-pound dumbbell arm curls, 5-pound dumbbell flyers, 5-pound dumbbell shoulder raises, etc. If the participant was unable to complete the exercise on their own, then they were able to hold onto the back of a chair with both hands to assist in completing the operation (standing lunge: the two seat backs are placed at the side of the body, and the backs of the chairs are used for the operation). Details of the exercises are shown in Table 2.

### 2.3. Testing Method

#### 2.3.1. Body Composition

Body composition was measured using an InBody 520 (Biospace Co., Ltd., Seoul, Korea). The InBody 520 estimates the body composition of the human body based on bioelectrical impedance analysis (BIA). The bioelectrical resistance method is useful in body composition research because the electrode that is in contact with the human body measures the resistance value (impedance) of the body with an electrical current. The participants climbed on the InBody, faced the front, and stood in an upright position for approximately 60 s. Finally, the body mass index (BMI), body fat percentage (BFP), basal metabolic rate (BMR), basal metabolic rate (BMR), and skeletal muscle mass (SMM) were determined from the results of the body composition analysis.

#### 2.3.2. Fitness Test

The senior fitness test (SFT) was developed based on the work of Rikli and Jones [27], as shown in Table 3. The purpose of the SFT test was threefold: (1) to determine if moderate-intensity physical activity can change body composition; (2) to determine whether moderate-intensity physical activity can improve upper and lower extremity muscle strength; and (3) to determine if moderate-intensity physical activity can improve agility and balance. The test items include body composition, 30 s chair stand, 30 s arm curl, 8-foot (2.44 m) up-and-go, 2 min step, and single leg, which are widely used in fitness tests for the elderly.

#### 2.3.3. Sarcopenia Test

Handgrip strength was assessed in the seated position. Participants sat comfortably in a standard chair with legs, a backrest, and fixed arms. The optimal grip distance was adjusted according to hand size, and the same chair was used for each measurement. Participants sat with their forearms resting on the arms of the chair and their wrists at the end of the arms of the chair. Wrists were in the neutral position with the thumbs up; the participants were encouraged to squeeze (with their arms placed 90 degrees to the side) for as long as they could or until the needle stopped rising using a Jamar hydraulic hand dynamometer (Model 5030; Sammons Preston, IL, USA). Additionally, gait speed (GS) was measured over a distance of 10 m and timed (>0.8 m/s). The purpose of this was to test the muscle strength in the lower limbs [2,28].

### 2.4. Statistical Analysis

Descriptive statistics were performed. Shapiro–Wilk tests were performed for all the dependent variables for both control and experimental groups and for both the pre-test and post-test data to check the normality assumption. The results showed that the hypothesis of normal distribution was supported for all the data. Descriptive statistics and *t*-tests were conducted to compare the pre-test and post-test of the experimental group, as well as post-test changes in body composition, upper- and lower-extremity muscle strength, balance ability, handgrip strength, and gait speed. Multiple regression analysis was used to evaluate sarcopenia. The significance level was set to *p* < 0.05. The Cohen’s d was calculated to determine the effect size of the *t*-tests and adequacy of the sample size [29]. Statistical analyses were performed using SPSS 20.0 software (IBM^®^, Armonk, NY, USA).

## 3. Results

### 3.1. Body Composition Testing

**Figure 1:**
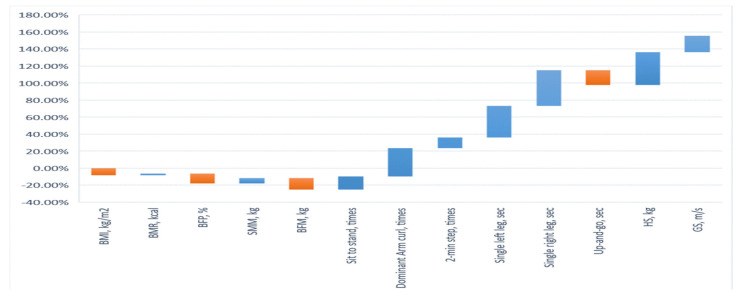
Participant outcomes after intervention with a moderately intensity physical activity program.

The test scores of the pre- and post-tests determining the body composition of the experimental group (EG) and control group (CG) are shown in Table 2 and Table 3. The results showed that the *t*-tests for the body composition in EG and CG failed to reach significant levels, suggesting homogeneity among the EG and CG participants. For the EG, the results of the *t*-test in terms of the pre- and post-tests determining body composition were significantly different. BMI decreased by 8.24% (*t* = 6.29, *p* < 0.05), BMR increased by 2.05% (*t* = −5.06, *p* < 0.05), BFP decreased by 11.57% (*t* = 8.48, *p* < 0.05), SMM increased by 6.23% (*t* = −8.03, *p* < 0.05), and BFM decreased by 13.66% (*t* = 5.12, *p* < 0.05). In addition, the post-test comparison results of the EG and CG all reached significance in terms of the BMI (*t* = −6.50, *p* < 0.05), BMR (*t* = 2.44, *p* < 0.05), BFP (*t* = −7.63, *p* < 0.05), SMM (*t* = 7.93, *p* < 0.05), and BFM (*t* =−5.27, *p* < 0.05). After the EG had undergone the prescribed moderate-intensity physical activity program, their overall body composition changed. In the EG, the BMI, BFP, and BFM decreased significantly, and BMR and SMM increased significantly. The results verified Hypothesis 1. The results are shown in Figure 1.

**Hypothesis** **1:** *Moderate-intensity physical activity can improve the body composition of the participants.*

### 3.2. Performance of Upper and Lower Extremity Muscle Strength

The results of the upper- and lower-extremity muscle strength of the EG and CG as measured by the SFT are shown in Table 2 and Table 3. The *t*-test on the post-test results in the EG showed that the participant strength for the 30 s sit-to-stand increased by 15.53% (*t* = −3.87, *p* < 0.05), the participant strength for the 30 s dominant arm curl increased by 33.45% (*t* = −6.58, *p* < 0.05), and the participant for the 2 min step increased by 12.50% (*t* = −3.34, *p* < 0.05). The results showed that the number of 30 s sit-to-stand motions increased, and the muscle strength of the lower limbs increased in the EG. The number of 30 s dominant arm curls increased, and the strength of the upper extremities increased in the EG group. The results confirmed Hypothesis 2. The results are shown in Figure 1.

**Hypothesis** **2:** *Moderate-intensity physical activity can improve the upper- and lower-extremity muscle strength of the participants.*

### 3.3. Performance of Balance Ability

The static and dynamic balance abilities of the EG and CG were examined by SFT, as shown in Table 2 and Table 3. The *t*-test on the pre- and post-test results of the EG showed that the time required for a single left leg increased by 37.01% (*t* = −6.65, *p* < 0.05); the time for a single right leg increased by 41.79% (*t* = −7.51, *p* < 0.05), and the time required for the 8-foot up-and-go was reduced by 17.13% (*t* = 7.09, *p* < 0.05). All of these increases were significant. After the moderate-intensity physical activity program, the static balance required for the single-leg stand was significantly improved in the EG, and the dynamic balance and agility required for the 8-foot up-and-go were significantly improved. The results validate Hypothesis 3. The results are shown in Figure 1.

**Hypothesis** **3:** *Moderate-intensity physical activity can improve balance ability in the participants.*

### 3.4. Analysis of Upper Limb Handgrip Strength and Lower Limb Gait Speed

The results of pre- and post-tests for upper limb handgrip strength and lower limb gait speed in the EG showed significant differences, indicating that the prescription of a moderate-intensity physical activity program effectively increased upper-extremity muscle strength (HS, *t* =−20.03, *p* < 0.05) and lower limb gait speed (GS, *t* =−16.55, *p* < 0.05) in the EG, as shown in Table 4 and Table 5. After the EG went through a moderate-intensity physical activity program, the average upper limb handgrip strength increased from 9.64 kg to 13.35 kg, an improvement of 38.49%; the average lower limb gait speed increased from 0.73 m/s to 0.87 m/s, an improvement of 19.18%. The results are shown in Figure 1.

### 3.5. Evaluated Analysis of Sarcopenia

Table 6 and Table 7 show the results of multiple regression model 1 and illustrate the body composition and the 30-s dominant arm curl test results to predict upper limb handgrip strength. The results are as follows: correlation coefficient R = 0.879, R^2^ = 0.773, adjusted R^2^ = 0.716, estimated standard error =0.945, R^2^ change =0.773, F change =53.905, F value change significance = 0.003. The overall multiple regression model reached a significant level, and the independent variables were tested by the post hoc test and were as follows: BMI β = 0.413, BMR β = 0.399, BFP β = 0.532, SMM β = 0.364, BFM β = 0.353, and dominant arm curl β = 0.541, indicating a fairly high related relationship between body composition and 30 s of dominant arm curls on hand grip strength. That is, the body composition values and the training prescription in this study were effective in evaluated the effectiveness of the program against upper-extremity sarcopenia.

Table 8 and Table 9 show the results of multiple regression model 2, in which the overall explanatory power of all of the independent variables for gait speed was 96.9%, and the adjusted R^2^ = 90.5%; the overall regression model was significant: F = 36.699, *p* = 0.001. The independent variables were tested by the post hoc test: BMI β = 0.352, BMR β = 0.316, BFP β = 0.340, SMM β = 0.540, BFM β = 0.398, sit-to-stand β = 4.757, 2 min step β = 6.831, single left leg β = 3.824, single right leg β = 3.833, and up-and-go β = 4.229, indicating that there was a correlation between body composition, sit to stand, 2 min step, single left leg, single right leg, up-and-go. That is to say, the training program that was prescribed in this can effectively evaluate of its effects in combating lower-extremity sarcopenia based on the participants’ body composition.

Summarizing the above multiple regression analysis, the elderly females involved in this study improved their body composition, upper- and lower-limb muscle strength, and balance ability after a moderate-intensity physical activity program and achieved significant improvements in their handgrip strength and gait speed, which also confirmed the enhancement of the upper- and lower-limb muscle strength. These results validate Hypothesis 4.

**Hypothesis** **4:** *Participants can optimize their upper and lower limb muscle strength and balance ability with this training program, and the program is able to evaluate sarcopenia.*

## 4. Discussion

The changes in the body composition of the women participating in the study as a result of upper and lower extremity muscle strength training were found to be consistent with our hypothesis, with participants experiencing significant decreases in BMI, BFP, and BMC through moderate-intensity physical activity. Experts believe that all physical activities can provide physical stimulation with different levels of intensity to produce different results [30]. In this study, the intensity of physical activity was defined. Specifically, when a person is at rest and watching TV, they consume one calorie per kilogram of body weight per hour (1 METs). As such, a 60 kg elderly woman sitting at work, watching TV, talking, or driving would consume only 60 calories per hour (60 METs). Hence, the intensity of physical activity in this study was defined as moderate intensity (3.0–5.9 METs) [24]. An elderly woman weighing 60 kg expends approximately 200 calories (5 METs × ⅔ h × 60) in one session of this physical activity program. Such activities will make people feel a little tired, have a slightly higher breathing and heart rate, and also sweat a little, which is also in line with many research findings suggesting that the intensity of physical activity to promote health should be more than moderate [31]. The results showed that these elderly females experienced a reduction in their average fat content and experienced weight loss after the completion of the moderate-intensity exercise program that was prescribed in this study. The results of the study showed that these elderly females experienced significant improvement in upper and lower extremity muscle strength after a moderately strenuous physical activity program, consistent with the findings of Rikli and Jones [27]. Mayer et al. demonstrated that older adults can increase their muscle mass and muscle strength through plyometric training at the intensities ranging from 60–85% of the participant’s personal maximum strength [32].

The results of this study found that the amount of time that the elderly females could spend standing on a single leg increased (the average improvement in the left leg was 37.01%, reaching 21.47 s; the average improvement in the right leg was 41.79%, reaching 18.24 s), and the dynamic balance speed of the 8-foot up-and-go increased (average improvement to 5.37 s), showing that the dynamic and static balance of the participants had improved after the completion of the moderate-intensity training program. These results are consistent with the studies of Zouita et al. and Rizzato et al. [33,34]. It is believed that training to improve muscle strength increases muscle strength and enhances the walking speed and balance ability of elderly females. Perez-Sousa et al. proved that a decrease in lower extremity muscle mass is correlated with a decrease in gait speed [35]. Wilkinson et al. confirmed that the middle-aged population starts to lose 1% of muscle mass every year, and elderly people who do not exercise for a long time will lose 50% by the age of 80–90 [3]. In recent years, it has been proven that the loss of muscle strength or function (walking speed, lifting heavy objects) can be the key feature of sarcopenia [36]. Symptoms of sarcopenia include walking slower than usual, difficulty lifting ordinary household objects, walking fatigue, and general weakness. Cho et al. confirmed that rapid muscle loss due to aging can be prevented through moderate intensity muscle training [17]. This research has also shown that progressive and regular muscle training is beneficial to improving balance ability. It has also been confirmed that increased balance in the elderly is associated with sarcopenia [37,38]. Therefore, in order to avoid the rapid decline of muscle strength caused by aging, it is recommended to increase the opportunities for physical activities of the elderly, such as cleaning, standing and walking freely, climbing stairs, and lifting groceries, which can improve the health function and quality of life of the elderly [19]. Fletcher et al. confirmed upper limb handgrip strength and lower limb walking speed can be used as a reference for evaluating sarcopenia [39]. The grip strength of the elder adult is much lower than that of the young. Average readings ranged from 6 to 8.6 kgf; the participant stood erect with her arm straight down by the side. This posture was recommended by Li and Yu [40]. Therefore, the participants (elderly females) in this study showed significant improvements in upper extremity handgrip strength and lower extremity walking speed in the post-test that was conducted after muscle training. The increase in muscle strength or muscle endurance adaptations in the extremities mainly occurred because these elderly females participated in higher intensity physical activities than usual [41]. Thus, according to this study, moderate-intensity trained elderly females experienced significant improvement in 30 s chair stand, 2 min step, single leg, and 8-foot up-and-go and 30 s of arm curls. That is, the overall skeletal muscle increased, the static balance became more stable, and the basal metabolic rate also improved in all of the participants, consistent with the findings of Sousa et al. [42].

Overall, from the results of this prediction study, it is possible to verify that a moderate-intensity physical activity program (exercise intensity of 3.0–5.9 METs) improves the body composition, upper and lower extremity muscle strength, and balance ability of elderly females and can effectively evaluate sarcopenia. The medical community recommends exercise as an effective way to improve sarcopenia [11,12,16,19,35,36,38]. Because exercise keeps the muscles active, it is the most effective way to fight sarcopenia. A combination of aerobic exercise, resistance training, and balance training can prevent and even reverse muscle loss.

## 5. Conclusions

Sarcopenia and geriatric fitness testing are important indicators of healthy aging in the elderly, and these parameters can be improved with appropriately designed physical activity programs. In this study, a moderately strenuous physical activity program designed for elderly females that lasted for eight weeks showed that body composition was optimized; balance, grip strength, and gait speed were effectively increased; and moderate intensity physical activity was effective for improving muscle strength and reducing sarcopenia.

## Figures and Tables

**Table 1:** Basic participant information.

Variable	CG (*n* = 15) M ± SD	EG (*n* = 15) M ± SD	*t*	*p*-Value
Age (years)	72.1 ± 5.7	71.8 ± 6.1	−0.74	0.591
Height (cm)	157.1 ± 8.2	157.8 ± 7.0	0.61	0.623
Weight (kg)	68.0 ± 13.5	68.5 ± 10.4	0.83	0.477

CG means control group, EG means experimental group.

**Table 2:** Moderately strenuous physical activity program.

Course	Posture and Motions	Operation Method
Cardiopulmonary function	Step-ups 20 (times) × 3 (set)	30 cm high steps: go up the steps with one foot, stand with both feet together, go down the steps with one foot, stand with both feet together and repeat.
Lower body strength	Chair squats 15 (times) × 3(set)	Place a chair behind you and start in the standing position; squat down and raise your hands horizontally, and then squat down and touch the chair with your buttocks and immediately rise into a standing position. Do this once.
	Pistol squat 10 (times) × 3 (set)	Stand on one foot and leave the other foot off the ground (you can hold a support) and perform 10 deep squats on the left and right feet.
	Standing lunges 20 (times) × 3 (set)	Start in a standing position; put your feet together. Step on one foot (with a stride of more than 60 cm), retract the leg that has been stepped out, and step out with the other foot. Repeat the motion with your left and right feet 20 times.
	Walk in place with high legs 20 (times) × 3 (set)	Start a standing position, raise the leg in place up to thigh-level, and repeat the operation with the left and right feet 20 times.
Upper body strength	5-pound dumbbell arm curls 12 (times) × 3(set)	Stand vertically with dumbbells in hand and perform flexion movements with both hands at the same time.
	5-pound dumbbell flyers 12 (times) × 3(set)	Stand vertically with dumbbells in your hands and abduct your hands to a horizontal level at the same time.
	5-pound dumbbell shoulder raises 12 (times) × 3(set)	Hold the dumbbells at shoulder height as the starting point, raise both hands vertically at the same time, and then return to the starting point.

**Table 3:** Brief description of SFT items.

Assessment Category	Test Item	Test Description
Lower body strength	30 s chair stand	Number of full stands in 30 s with arms folded across chest
Upper body strength	30 s arm curl	Number of bicep curls in 30 s holding a hand weight (women’s 5 lb)
Aerobic endurance	2 min step	Number of full steps completed by raising each knee to point midway between the patella and iliac crest (number of times knee reaches target) in 2 min
Static balance	Single leg (SL)	Participants must lift one leg off the ground and maintain their balance while standing
Dynamic balance	8-foot up-and-go	Number of seconds required to get up from a seated position, walk 8 feet (2.44 m), turn around, and return to a seated position on the chair

Description of senior fitness test (SFT) items from Rikli and Jones [27].

**Table 4:** Body composition, SFT test, and sarcopenia scores of the EG and CG for the pre- and post-tests.

Variables	CG (*n* = 15)		EG (*n* = 15)		Improvement (%)
	Pre	Post	Pre	Post	
BMI, kg/m^2^	27.29 ± 2.41	27.38 ± 2.48	27.30 ± 2.14	25.22 ± 2.00	−8.24
BMR, kcal	1207 ± 66.42	1213 ± 72.67	1220 ± 94.0	1245 ± 101.8	2.05
BFP, %	32.19 ± 5.20	32.43 ± 5.56	32.30 ± 5.25	28.95 ± 4.01	−11.57
SMM, kg	26.23 ± 2.52	26.01 ± 2.50	26.69 ± 2.00	28.35 ± 2.25	6.23
BFM, kg	21.79 ± 1.99	21.64 ± 1.97	21.80 ± 2.08	19.18 ± 2.07	−13.66
Sit to stand, times	18.93 ± 2.89	19.00 ± 3.05	18.93 ± 3.13	21.87 ± 2.90	15.53
Dominant arm curl, times	20.4 ± 3.00	20.27 ± 2.69	20.33 ± 3.17	27.13 ± 3.64	33.45
2 min step, times	113.2 ± 7.55	112.87 ± 6.25	113.13 ± 6.92	127.27 ±8.55	12.50
Single left leg, sec	15.76 ± 7.36	15.51 ± 6.58	15.67 ± 9.39	21.47 ± 8.99	37.01
Single right leg, sec	12.91 ± 6.91	12.95 ± 6.49	12.85 ± 7.76	18.24 ± 7.47	41.79
Up-and-go, sec	6.19 ± 0.99	6.24 ± 0.94	6.29 ± 1.63	5.37 ± 0.90	−17.13
HS, kg	9.66 ± 0.71	9.65 ± 0.69	9.64 ± 0.61	13.35 ± 1.16	38.49
GS, m/s	0.74 ± 0.05	0.73 ± 0.05	0.73 ± 0.05	0.87 ± 0.09	19.18

EG—experimental group; CG—control group; BMI—body mass index; BMR—basal metabolic rate; BFP—body fat percentage; SMM—skeletal muscle mass; BCM—body cell mass; SFT—senior fitness test; AC—arm curl; SL—single leg; HS—handgrip strength; GS—gait speed; kg/m^2^—kilograms per squared meters; m/s—meters per second. Values are presented as means ± standard deviations.

**Table 5:** *T*-test of the body composition results of the EG and CG as well as for the SFT, HS, and GS pre- and post-tests.

Variables	CG-Pre	EG-Pre	EG-Pre	EG-Post
	CG-Post	CG-Pre	EG-Post	CG-Post
BMI, kg/m^2^	−1.40	0.64	6.29 *	−6.50 *
BMR, kcal	−1.57	1.01	−5.06 *	2.44 *
BFP, %	−1.51	0.383	8.48 *	−7.63 *
SMM, kg	1.78	1.03	−8.03 *	7.93 *
BFM, kg	1.01	0.42	5.12 *	−5.27 *
Sit to stand, times	0.24	0.01	−3.87 *	4.01 *
Dominant arm curl, times	−0.20	−0.20	−6.58 *	6.15 *
2 min step, times	0.53	−0.04	−3.34 *	3.04 *
Single left leg, sec	−0.11	−0.08	−6.65 *	6.21 *
Single right leg, sec	0.54	−0.05	−7.51 *	5.50 *
Up-and-go, sec	−1.48	1.03	7.09 *	−7.70 *
HS, kg	0.135	−0.11	−20.03 *	8.64 *
GS, m/s	2.18	−0.32	−16.55 *	9.43 *

* *p* < 0.05. EG—experimental group; CG—control group; BMI—body mass index; BMR—basal metabolic rate; BFP—body fat percentage; SMM—skeletal muscle mass; BFM—body fat mass; HS—handgrip strength; GS—gait speed; kg/m^2^—kilograms per squared meters; m/s—meters per second. Values are presented as independent sample *t*-test.

**Table 6:** Summary of the HS results in multiple regression model 1.

Model	R	R^2^	Adjusted R^2^	Estimated Standard Error	R^2^ Change	F Change	Sig
1	0.879 (a)	0.773	0.716	0.945	0.773	53.905 *	0.003

* *p* < 0.05; (a) refers to the predictor variables of this study, including constant, BMI, BMR, BFM, BFP, SMM, dominant arm curl, etc.

**Table 7:** HS coefficients in multiple regression model 1.

Model	Unstandardized Coefficients		Standardized Coefficients	*t*	Sig
	B	Std. Error	Beta		
(Constant)	5.513	0.613	-	7.342 *	0.000
BMI	0.224	0.030	0.413	6.718 *	0.000
BMR	0.212	0.006	0.399	6.289 *	0.000
BFP	0.318	0.001	0.532	7.163 *	0.000
SMM	0.244	0.060	0.364	5.898 *	0.008
BFM	0.242	0.055	0.353	4.914 *	0.017
Dominant arm curl	0.329	0.082	0.541	7.329 *	0.000

* *p* < 0.05.

**Table 8:** GS results for multiple regression model 2.

Model	R	R^2^	Adjusted R^2^	Estimated Standard Error	R^2^ Change	F Change	Sig
2	0.984 (a)	0.969	0.905	0.029	0.969	36.699 *	0.001

* *p* < 0.05; (a) refers to the predictor variables of this study, including constant, BMI, BMR, BFM, BFP, SMM, sit-to-stand, 2 min step, single left leg, single right leg, up-and-go, etc.

**Table 9:** GS coefficients for multiple regression model 2.

Model	Unstandardized Coefficients		Standardized Coefficients	*t*	Sig
	B	Std. Error	Beta		
(Constant)	6.637	0.715		6.058 *	0.000
BMI	0.218	0.034	0.352	4.039 *	0.016
BMR	0.203	0.046	0.316	3.221 *	0.037
BFP	0.212	0.038	0.340	3.791 *	0.029
SMM	0.248	0.022	0.540	7.048 *	0.000
BFM	0.232	0.027	0.398	5.093 *	0.003
Sit to stand	0.225	0.031	0.377	4.757 *	0.007
2 min step	0.233	0.025	0.415	6.831 *	0.000
Single left leg	0.209	0.039	0.338	3.824 *	0.025
Single right leg	0.213	0.036	0.342	3.833 *	0.019
Up-and-go	0.216	0.029	0.357	4.229 *	0.011

* *p* < 0.05.

## Data Availability

The experimental results used real data obtained from the study participants before and after the measurement data obtained after the training program. The participants agreed with the data structure via a confirmation, and confirmation can be disclosed with reasonable availability. All of the datasets on which the conclusions of the paper rely are available to editors, reviewers, and readers.
